# Facial affect processing deficits in schizophrenia: A meta-analysis of antipsychotic treatment effects

**DOI:** 10.1177/0269881114560184

**Published:** 2015-02

**Authors:** Anthony S Gabay, Matthew J Kempton, Mitul A Mehta

**Affiliations:** 1 Department of Neuroimaging, Institute of Psychiatry, Psychology and Neuroscience, King’s College London, London, UK; 2 Department of Psychosis Studies, Institute of Psychiatry, Psychology and Neuroscience, King’s College London, London, UK

**Keywords:** Affect, antipsychotic agents, meta-analysis, schizophrenia

## Abstract

Social cognition, including emotion processing, is a recognised deficit observed in patients with schizophrenia. It is one cognitive domain which has been emphasised as requiring further investigation, with the efficacy of antipsychotic treatment on this deficit remaining unclear. Nine studies met our criteria for entry into a meta-analysis of the effects of medication on facial affect processing, including data from 1162 patients and six antipsychotics. Overall we found a small, positive effect (Hedge’s g = 0.13, 95% CI 0.05 to 0.21, *p* = 0.002). In a subgroup analysis this was statistically significant for atypical, but not typical, antipsychotics. It should be noted that the pooled sample size of the typical subgroup was significantly lower than the atypical. Meta-regression analyses revealed that age, gender and changes in symptom severity were not moderating factors. For the small, positive effect on facial affect processing, the *clinical* significance is questionable in terms of treating deficits in emotion identification in schizophrenia. We show that antipsychotic medications are poor at improving facial affect processing compared to reducing symptoms. This highlights the need for further investigation into the neuropharmacological mechanisms associated with accurate emotion processing, to inform treatment options for these deficits in schizophrenia.

## Introduction

Antipsychotic medication is used to treat positive symptoms in schizophrenia (National Institute for Health and Care Excellence, 2014). However, deficits in social cognition have been shown to be strongly associated with functional outcome (Green et al., 2004), and is one of eight domains identified by the initiative ‘Measurement and Treatment Research to Improve Cognition in Schizophrenia’ (MATRICS), which require further investigation and treatment strategies (Nuechterlein et al., 2004).

In a review of the literature, Kucharska-Pietura and Mortimer (2013) concluded that antipsychotics are unlikely to facilitate the recovery of social cognition deficits in schizophrenia based on a review of 15 articles. By far the most widely studied aspect of social cognition is emotion processing, which is typically assessed using tasks requiring participants to perceive, identify and discriminate between facial emotion expressions. A deficit in these abilities has consistently been found in schizophrenia (Kohler et al., 2010). In a review specific to the facial affect recognition literature, Hempel and colleagues concluded, based on eight studies, that antipsychotic medication does not successfully treat this aspect of schizophrenia (Hempel et al., 2010).

While these reviews provide valuable descriptions of the relevant literature, they are unable to provide a *quantitative* analysis of the effects of antipsychotic medication on these cognitive deficits. It also remains possible that the effects of treatment may be small, or affected by moderating factors such as age, gender or type of medication. In order to address these questions we have performed a meta-analysis of studies specifically investigating the effects of antipsychotics on emotion processing in schizophrenia.

## Methods

**Figure 1.**
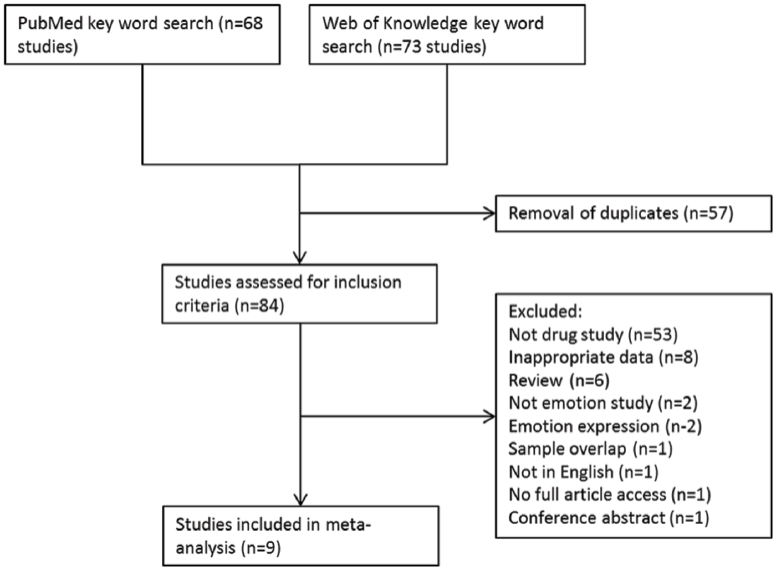
Flowchart showing study selection for the meta-analysis.

A literature search was carried out using PubMed and Web of Knowledge databases, entering the search terms ‘schizophrenia AND facial AND emotion AND antipsychotic’ in May 2014. In addition, a manual search was carried out in reference sections of papers returned. We included English-language studies that: (a) used a task investigating facial emotion processing; (b) specifically investigated the effects of antipsychotic medication; (c) provided pre- and post-medication data; and (d) included patients with a diagnosis of schizophrenia. Nine studies met these inclusion criteria, confirmed by two of the authors (ASG and MAM; see Figure 1).

Studies employed a range of tasks, with the predominant outcome measure being number of correct/incorrect responses (*n* = 8). The outcome measure from one study (Cabral-Calderin et al., 2010) was number of phases of facial morphing before a correct response. Where data were not available in an appropriate form, authors were contacted requesting additional information.

For studies investigating multiple antipsychotics, each drug was entered into the meta-analysis separately. These were independent samples. Hedge’s g and its 95% confidence intervals (CI) were calculated for each study and each drug. Hedge’s g is a measure of effect size similar to Cohen’s d, but corrected for small sample size (Ellis, 2010). A random effects meta-analysis, subgrouped by typical and atypical antipsychotics, was carried out using Review Manager 5.2 (The Nordic Cochrane Centre, 2012), using an inverse variance weighted model. Between-study heterogeneity was assessed using the I^2^ statistic. Egger’s intercept and visual inspection of funnel plots was used to assess evidence of publication bias (Egger et al., 1997). We also carried out meta-regression analyses using the metareg module in Stata Statistical Software (Harbord and Higgins, 2008; StataCorp, 2011) to assess the influence of symptoms, age and gender on task performance.

One study (Cabral-Calderin et al., 2010) reported subscales of tasks with no overall score. The total score and standard deviation (SD) for these subscales were calculated, using an estimation of the correlation coefficient between subscales as 0.8 in order to sum the SDs. Sensitivity analyses were carried out to determine if altering this estimation affected the pooled effect size.

## Results

### Literature search

After 57 duplicates were removed, 84 studies were returned by the original search. Of these, nine studies met the inclusion criteria, investigating six antipsychotics (haloperidol, perphenazine, perazine, riseridone, quetiapine, olanzapine) (Bediou et al., 2007; Behere et al., 2009; Cabral-Calderin et al., 2010; Daros et al., 2014; Harvey et al., 2006; Lewis and Garver, 1995; Penn et al., 2009; Sergi et al., 2007; Wölwer et al., 1996) in 1152 patients with schizophrenia (see Table 1 for study details). Overall there was no bias with regard to which emotions were examined. Two studies (Daros et al., 2014; Harvey et al., 2006) only focused on two emotions – the range of intensity from very happy to very sad; four studies (Behere et al., 2009; Cabral-Calderin et al., 2010; Lewis and Garver, 1995; Wölwer et al., 1996) investigated processing of happiness, disgust, sadness, surprise, anger and fearful expressions; one study (Bediou et al., 2007) investigated processing of neutral, happiness, disgust, fear and anger; two studies investigated processing of happiness, sadness, anger, surprise, fear and shame (Penn et al., 2009; Sergi et al., 2007).

**Table 1.** Details of included studies.

Study first author	Date	Drug	Dosage mg/day	Study design	N	Sex (% M)	Mean age (+/−ve: positive/negative symptom scale)	Symptom severity			Duration of illness (years)	Time from baseline to follow-up (weeks)	Task (R = recognition, D = discrimination)
								Measure	Baseline	Endpoint			
Lewis	1995	Haloperidol	5–20	D, P, FL	18	No data	38.9	BPRS	45.7	40.2	Not given	2	FAR (R)
Wölwer	1996	Haloperidol Perazine	531±313 436±217 CPZE	W, P, FL	12 20	67 70	33.2 31.8	(Across all patients) BPRS (+ve) (−ve) SANS	25.72(8.13) 10.84(3.14) 13.63(5.00)	17.61(4.77) 9.53(3.42) 11.56(4.52)	6.7 ± 6.9	4	FAR (R)
Bediou	2007	Haloperidol	10 (1.6)	P, D, FL	26	92	24.3	(Includes non-completers) PANSS (+ve) (−ve) PANSS(general)	29.5(7.1) 27.2(7.6) 39(6.6)	10.2(6.7) 11.4(3.8) 20(4)	First episode	4.3 (mean)	EFER (R)
Sergi	2007	Haloperidol Olanzapine Risperidone	8 15 4	P, DB, R, FD	20 40 40	100 86 87	50 49.2 48.2	BPRS (+ve) (−ve) (+ve) (−ve) (+ve) (−ve)	Not given	3.0 (0.9) 2.1 (0.8) 2.5 (1.0) 2.3 (0.8) 2.8 (1.0) 2.3 (0.8)	Not given	8	FEIT (R)
Behere	2009	Risperidone	4	P, D, FD	25	70	29.4	SANS SAPS	60.2(25.1) 29.3(13.6)	43.2(13.1) 12.6(12.1)	1.4 (1.5)	5.5 (mean)	TRENDS (R)
Harvey	2006	Quetiapine Risperidone	529.62(288.28) 5.33(2.13)	P, DB, R, FL	124 142	78 76	40.2 39.9	PANSS (+ve) (−ve) (total) (+ve) (−ve) (total)	16.77(6.56) 17.27(5.95) 22.52(22.10) 17.66(5.54) 18.58(5.62) 71.09(20.76)	Not given	Not given	8	PEAT (intensity)
Penn	2009	Perphenazine Olanzapine Quetiapine Risperidone	8 7.5 200 1.5	P, R, DB, FL, W	159 170 161 161	75 (overall)	41.0 (overall)	Across all patients PANSS (total)	74.29(17.48)	Not given	14.49 (10.92)	8	FEDT (D)
Cabral-Calderin	2010	Quetiapine	413.5 (165.6)	P, W, FL	34	56	35	PANSS (+ve) (−ve) (general) (total)	15.58(7.16) 16.5(7.94) 34.32(12.55) 66.41(22.83)	12.76(5.81) 14.23(7.29) 28.23(10.24) 55.23(18.96)	9.22 (8.54)	12	EEMT (R)
Daros	2014	Risperidone	3.53 (1.8)	P, W, FL	19	79	21.5	PANSS (+ve) (−ve) (total)	24.74(4.41) 19.26(5.67) 80.11(15.47)	15.61(4.96) 15.61(6.21) 61.61(16.71)	First episode		PEAT (intensity)

P: pre-post design; D: drug-free baseline; R: randomised; W: washout/drug cross-over period; DB: double-blind; FD: fixed-dose; FL: flexible dose; FAR: facial affect recognition (Ekman and Friesen, 1976); EFER: emotional facial expression recognition; FEIT: facial emotion identification test, photos developed by Izard (1971) and Ekman and Friesen (1976); TRENDS: Tool for Recognition of Emotions in Neuropsychiatric DisorderS (Behere, 2009); PEAT: Penn Emotional Acuity Test (Cornblatt, 1989); FEDT: Face Emotion Discrimination Test (Kerr and Neale, 1993); EEMT: Emotional Expression Multimorph Task (Mendoza, 2011); CPZE: Chlorpromazine equivalents; BPRS: Brief Psychiatric Rating Scale; SANS: Scale for the Assessment for Negative Symptoms; SAPS: Scale for the Assessment for Positive Symptoms; PANSS: Positive and Negative Syndrome Scale.

### Overall meta-analysis

**Figure 2.**
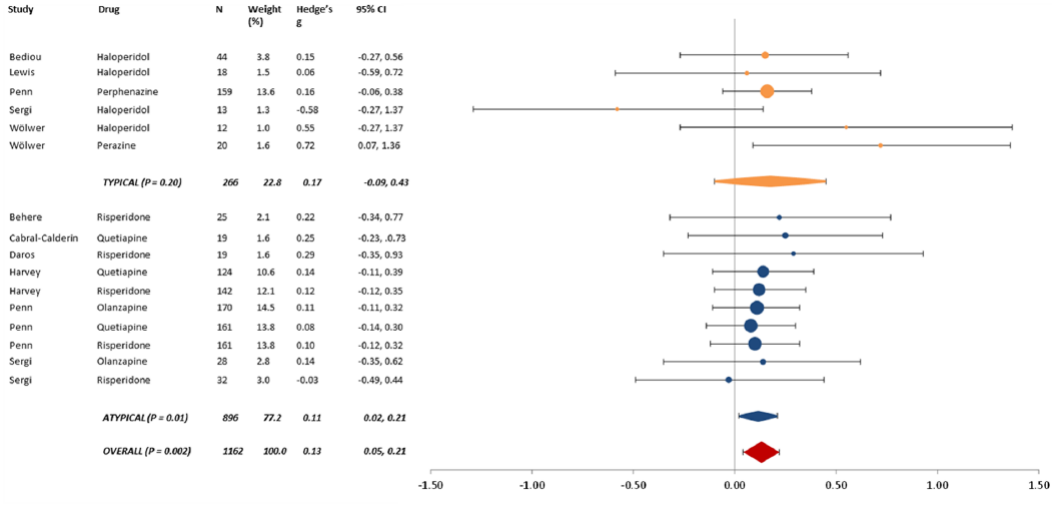
Results of meta-analysis. Data identified by study first author and antipsychotic.

The overall pooled Hedge’s g was 0.13 (95% CI 0.05 to 0.21, *p* = 0.002) (see Figure 2). There was no significant overall between-study heterogeneity (*p* = 0.85), and no evidence of publication bias (*p* = 0.49).

Changing the estimate of the correlation coefficient when summing SDs across subscale data from Cabral-Calderin et al. (2010) had no effect on the overall pooled effect size.

### Subgroup analyses

There was no statistically significant effect when the analysis was restricted to typical antipsychotics (Hedge’s g = 0.17, 95% CI -0.09 to 0.43, *p* = 0.16). This group showed no significant between-study heterogeneity (*p* = 0.16), and no evidence of publication bias (*p* = 0.95). This analysis included data from 266 participants.

When the analysis was restricted to atypical antipsychotics, the pooled Hedge’s g was statistically significant, at 0.11 (95% CI 0.02 to 0.21, *p* = 0.01). There was no significant between-study heterogeneity (*p* = 1.0), and no evidence of publication bias (*p* = 0.15). This analysis included data from 896 participants.

### Meta-regression

We carried out meta-regression analyses to assess the influence of age, gender, duration of treatment, and change in positive and negative symptoms, on the effect size. We were unable to obtain a breakdown of age and gender data across drugs for one study (Penn et al., 2009), and gender data from another (Lewis and Garver, 1995). From the nine studies, we were able to obtain pre and post symptom scores from only five, comprising data from 388 patients (the overall effect size for facial affect processing remained significant for this subset of studies, Hedge’s g = 0.15, *p* = 0.05). With the exception of one study (n = 26; Bediou et al., 2007), these data came from studies investigating atypical antipsychotics (Behere et al., 2009; Cabral-Calderin et al., 2010; Daros et al., 2014; Harvey et al., 2006). Four of these five studies reported pre- and post-Positive and Negative Symptom Scale (PANSS) (positive and negative symptom scales) data, while one reported data for the Scale for the Assessment of Negative Symptoms (SANS) and Scale for the Assessment of Positive Symptoms (SAPS). Percentage change in symptom scores were entered into the meta-regression, thus making these two scales comparable.

The meta-regression analyses suggest that neither age nor gender act as a moderator of effect size (*p =* 0.13 and *p =* 0.49, respectively). Furthermore, duration of treatment did not act as a moderator of effect size (*p* = 0.48). In addition, changes in positive and negative symptoms were not moderators (*p =* 0.83 and *p =* 0.97, respectively). That is to say, although these studies did report an improvement in both positive and negative symptoms from baseline to follow-up, the analyses suggest that the observed change in overall effect size for facial affect processing is independent of this symptom change.

## Discussion

We present data from the first meta-analysis of the effects of antipsychotic medication on emotion processing deficits in schizophrenia. We found a small, positive effect on facial affect processing tasks (Hedge’s g = 0.13). Subgroup analyses suggest that this positive effect is largely driven by atypical rather than typical antipsychotics. However, given the smaller sample size of the typical subgroup, we cannot rule out the possibility that there was not enough statistical power to identify the small effect in this group.

It is important to note that the overall effect size is particularly small. In a meta-analysis of facial affect identification deficits in schizophrenia, Kohler and colleagues reported a Cohen’s *d* of -0.89, rising to -1.41 when restricted to unmedicated patients (Kohler et al., 2010). Thus, it is questionable whether the effect we found in the current analysis would be *clinically* significant in terms of treating deficits in emotional function. Indeed, a recent multiple-treatments meta-analysis of the efficacy of 15 antipsychotics showed Hedge’s g ranging from -0.33 to -0.88 (median -0.44) for reducing symptoms compared to placebo (Leucht et al., 2013). It is clear that in comparison, antipsychotic medications are poor at improving facial affect processing deficits. Therefore, it is important to establish the neural mechanisms by which these deficits occur, as well as the small improvements seen with existing treatments, in order to inform better pharmacological targets.

The beneficial effect of antipsychotics on positive symptoms is believed to be due to their antagonistic action at dopamine D2 receptors (Seeman, 2004). It has been argued that dopamine plays an important role in emotion processing and recognition, and that emotion processing deficits in schizophrenia are associated with altered activity in the amygdala and prefrontal cortex (PFC) (Salgado-Pineda et al., 2005). Evidence suggests that individual differences in performance during processing of emotionally-relevant stimuli are associated with two different polymorphisms related to the dopamine D2 receptor gene (Blasi et al., 2009; Peciña et al., 2013). These are linked to differences in activity in the amygdala, PFC and anterior cingulate cortex. Thus, the dopaminergic effect of antipsychotic medication may play a role in the small changes in facial affect processing seen in the present study.

Stip and colleagues (Stip et al., 2005) provide data that suggest that treatment with quetiapine improves emotion processing in schizophrenia patients with blunted affect, and that this improvement is associated with modulation of neural activity in the PFC. Conversely, studies using antipsychotic medication in healthy participants have suggested that D2 antagonism impairs facial affect processing (Gibbs et al., 2010; Lawrence et al., 2002), although the medications used in those studies (sulpiride and amisulpride) were not represented in the sample of studies included in the current meta-analysis. These results highlight the subtleties of dopamine D2 receptor involvement in affective processing.

Other mechanisms by which antipsychotics may have an effect on facial effect processing are via serotoninergic action. Serotonin has been implicated as being key to emotion processing in a number of studies (e.g. Browning et al., 2007; Chen et al., 2008; Fu et al., 2007; Hornboll et al., 2013). These studies have largely involved administration of selective serotonin reuptake inhibitors (SSRIs) to healthy individuals, as well as in depression studies. The serotonin 2A receptor (5-HT2A) has particularly been associated with alterations in emotion processing, as shown in studies investigating facial affect processing using ketanserin, a 5-HT2A receptor antagonist (Hornboll et al., 2013; Kometer et al., 2012). Serotonergic action could explain the difference in efficacy between typical and atypical antipsychotics, as many atypicals act on 5-HT2A receptors.

There are surprisingly few pharmacological studies specifically investigating the effects of medication on facial affect processing, and emotion processing as a whole in schizophrenia. As such, the scope of the present analysis is restricted to the nine studies returned by the literature search. However, these studies included a combined total of 1162 patients. It is the nature of meta-analyses that one is limited by the data available, and by the design of the studies included. Some of the included studies used a naturalistic approach to dosage, and only three (Harvey et al., 2006; Penn et al., 2009; Sergi et al., 2007) were double-blind, randomised-control studies. Despite this variability in study design there was no statistically significant heterogeneity seen in the meta-analysis, increasing confidence in its findings. Also, in this meta-analysis all emotional expressions were pooled. Although this may add additional heterogeneity, this was necessary due to the relatively small number of studies available. Ethical and practical considerations limit the use of placebo-controlled studies in patients with schizophrenia and so direct comparisons of medication and placebo within patient groups was not possible. Furthermore, additional analyses investigating how changes in facial affect processing varied with other cognitive processing measures would be useful. However, few of the included studies reported such measures, and for those that did there was inconsistency in the scales used. Meta-regression analysis assessing the potential modulatory effect of duration of illness may also have been informative. Unfortunately this information was not broken down by medication in a sufficient number of included studies for such an analysis to be carried out. Finally, it should be noted that, as all of the studies used a pre-post design, the effects of learning cannot be ruled out.

## Conclusion

This study presents the first meta-analysis of the effects of antipsychotic medication on facial affect processing. We found a small, positive effect of antipsychotics, substantially lower than both the size of the typical deficit seen in schizophrenia and the efficacy for symptoms reduction, questioning the likely clinical significance. Subgroup analyses suggest the small positive effect is driven by atypical rather than typical antipsychotics, although the difference between the two treatment classes was not significant. Given the small effect size it is important that research continues to investigate the neural and neuropharmacological mechanisms associated with accurate emotion processing, in an attempt to inform further treatment options for these deficits in schizophrenia and other affective disorders.
